# Rationale and design of the Pan-African Sudden Cardiac Death survey: the Pan-African SCD study

**DOI:** 10.5830/CVJA-2014-035

**Published:** 2014

**Authors:** Aimé Bonny, Aimé Bonny, Marcus Ngantcha, Sylvie Ndongo Amougou, Adama Kane, Sonia Marrakchi, Emmy Okello, Georges Taty, Abdulrrazzak Gehani, Mamadou Diakite, Mohammed A Talle, Pier D Lambiase, Martin Houenassi, Ashley Chin, Harun Otieno, Gloria Temu, Isaac Koffi Owusu, Kamilu M Karaye, Abdalla AM Awad, Bo Gregers Winkel, Silvia G Priori, Silvia G Priori

**Affiliations:** Teaching Hospital Laquintinie, University of Douala, Douala, Cameroon; Service de Cardiologie, Centre Hospitalier Victor Provo, Roubaix, France; Biostatistics, Statprest, Paris, France; Service de Réanimation Médicale, Centre Hospitalier, Universitaire de Yaoundé, Cameroon; Service de Cardiologie, Centre Hospitalier le Dantec, Dakar, Senegal; Service de Cardiologie, Hopital Abderrrahmen Mami Ariana, Tunis, Tunisia; Department of Internal Medicine, Mulago Hospital, Makerere University, Kampala, Uganda; Service de Médecine Interne, Centre Hospitalier Général de Port-Gentil, Gabon; Heart Hospital, HMC, Tripoli, Libya; Service de Cardiologie, Hôpital Général de Bamako, Mali; Department of Internal Medicine, University of Maiduguri Teaching Hospital, Nigeria; Institute of Cardiovascular Sciences, University College London, UK; Service de Cardiologie, Centre Hospitalier Universitaire Abomey Calavi de Cotonou, Benin; Department of Cardiology, UCT Private Academic Hospital, Cape Town, South Africa; Department of Cardiology, Aga Khan University Hospital, Nairobi, Kenya; Department of Cardiology, Kilimanjaro Christian Medical Centre, Tanzania; Department of Cardiology, University Teaching Hospital of Accra, Ghana; Department of Cardiology, Aminu Kano Teaching Hospital, Kano, Nigeria; Department of Cardiology, University Hospital of Khartoum, Sudan; Department of Cardiology, Rigshospitalet, Copenhagen, Denmark; Department of Molecular Genetics, Fondazione Salvatore Maugeri, IRCCS, Pavia, Italy; Department of Molecular Medicine, University of Pavia, Italy

**Keywords:** sudden cardiac death, epidemiology, ethnicity, Africa

## Abstract

**Background:**

The estimated rate of sudden cardiac death (SCD) in Western countries ranges from 300 000 to 400 000 annually, which represents 0.36 to 1.28 per 1 000 inhabitants in Europe and the United States. The burden of SCD in Africa is unknown. Our aim is to assess the epidemiology of SCD in Africa.

**Methods:**

The Pan-Africa SCD study is a prospective, multicentre, community-based registry monitoring all cases of cardiac arrest occurring in victims over 15 years old. We will use the definition of SCD as ‘witnessed natural death occurring within one hour of the onset of symptoms’ or ‘unwitnessed natural death within 24 hours of the onset of symptoms’. After appro val from institutional boards, we will record demographic, clinical, electrocardiographic and biological variables of SCD victims (including survivors of cardiac arrest) in several African cities. All deaths occurring in residents of districts of interest will be checked for past medical history, circumstances of death, and autopsy report (if possible). We will also analyse the employment of resuscitation attempts during the time frame of sudden cardiac arrest (SCA) in various patient populations throughout African countries.

**Conclusion:**

This study will provide comprehensive, contemporary data on the epidemiology of SCD in Africa and will help in the development of strategies to prevent and manage cardiac arrest in this region of the world.

## Abstract

For many years there has been a debate about the definition and nature of ‘sudden death’ or out-of-hospital cardiac arrest.^1^-^8^ Issues pertaining to this debate have been the temporal definition of ‘sudden’, whether death was unexpected, whether death was witnessed, and the aetiology of the event. The time frame used to describe the duration of the terminal event initially was 24 hours. The current definition of sudden cardiac death (SCD) describes death within one hour of the onset of symptoms,^2^ since this period seems to describe most accurately patients with arrhythmic sudden cardiac death.^9^

A very difficult issue is the classification of unwitnessed deaths. Most authors have erred in favour of classifying such events as SCDs, even though it is often impossible to determine when the patient was last seen alive or the duration of symptoms prior to death. Hence, SCD can be defined as follows: ‘Natural death due to cardiac causes, heralded by abrupt loss of consciousness within one hour of the onset of acute symptoms’. Pre-existing heart disease may have been known to be present, but the time and mode of death are unexpected.^1^

The incidence of SCD occurring out of hospital varies with age, gender and presence or absence of cardiovascular disease. Incidence rates of SCD between 0.36 and 1.28 per 1 000 inhabitants per year have been reported in Europe and the United states.^10^-^13^ In these studies, only witnessed victims seen or resuscitated by the emergency medical services are included; these data therefore underestimate the incidence of SCD in the general population. Sudden cardiac death is responsible for about 300 000 to 400 000 deaths per year in Europe and the United States, respectively.^14^

Several diseases linked with sudden cardiac arrest (SCA) have been reported.^15^,^16^ Autopsy studies in unselected subjects suggest that about two-thirds of such deaths are cardiac in origin, with coronary artery disease and its complications accounting for the overwhelming majority of deaths in the industrialised world.^17^,^18^ Indeed, coronary artery disease (CAD) is the leading cause of sudden death worldwide.^3^

In Europe, cardiovascular diseases (CVD) account for around 40% of all deaths under the age of 75 years. SCA is responsible for more than 60% of adult deaths from ischaemic heart disease (IHD).^2^ Conversely, in young populations under 40 years, inherited ‘arrhythmogenic’ cardiac disorders are the main cause.^8^ The initial recorded rhythm in patients presenting with a sudden cardiovascular collapse is ventricular fibrillation (VF) in 75 to 80%, whereas bradyarrhythmias and asystole are thought to contribute to a minority of SCDs.^4^,^16^

## Rationale

The high prevalence and incidence of SCD in Western countries have led to the recognition that SCD is a major public health problem and the increased deployment of automatic external defibrillators in public places.

Recent reports by the World Health Organisation (WHO) indicated that non-communicable diseases (NCDs) are becoming a significant cause of morbidity and mortality in African countries.^19^-^26^ About 50% of this burden is attributable to CVD.^27^ Projections from the Global Burden of Disease project suggest that from 1990 to 2020, the burden of CVD faced by African countries will double and a large proportion of the victims of CVD will be middle-aged people.^28^ National public health policies regarding detection, prevention and treatment of NCDs are inconsistent, mainly due to lack of epidemiological data.

Regarding the magnitude of the problem, the ministers of health and heads of delegations of the WHO African region convened at a regional consultation on the prevention and control of NCDs in Brazzaville and acknowledged the everincreasing dual burden of communicable and non-communicable diseases in the region, and the associated disabilities and premature deaths.^29^ Almost all leading causes of SCD have been described in Africa (Table 1). Indeed some reports of SCD in sub-Saharan Africa have been published;^30^-^37^ and several studies report conflicting data regarding the prevalence of CHD in sub-Saharan Africa.^38^-^40^

**Table 1:** Reported cases of syncope or sudden cardiac death in black Africans living in Africa

	*Main disease*	*Authors*	*Title*	*Country*	*Type of publication*	*Journal and year*	*Size (patients)*
1	BrS	Bonny A, *et al*.	Brugada syndrome in pure black Africans	Ivory Coast, Benin, RD of Congo	Article	*Journal of Cardiovascular Electrophysiology 2008*	6
		Ouali S, *et al*.	Clinical and electrophysiological profile of Brugada syndrome in the Tunisian population	Tunisia	Article	*Pacing and Clinical Electrophysiology* 2011	24
2	HCM	Hiam I, *et al*.	Mort subite du sportif au Sénégal: étude rétrospective sur 8 ans	Senegal	Abstract	PASCAR conference, Dakar 15–20 May, 2013	5
3	IHD	Rotimi O, *et al*.	Sudden unexpected death from cardiac causes in Nigerians: a review of 50 autopsied cases	Nigeria	Article	*International Journal of Cardiology *1998	50
4	Paediatric sample	Arthur JT, *et al*.	Sudden deaths: cardiac and non-cardiac in children in Accra	Ghana	Article	*West Africa J *1995	16
5	CAD and RHD	Schneider J, *et al*.	Causes of sudden death in Addis Ababa, Ethiopia	Ethiopia	Article	*Ethiop Med J* 2001	63
7	Unknown	Houenassi M, *et al*.	Aspect epidémiologiques de la mort subite dans la ville de Parakou	Benin	Abstract	PASCAR conference, Dakar 15–20 May, 2013	23
8	Multiple causes (CAD, DCM, LQTS, RHD)	Talle MA, *et al*.	SCD in sub-Saharan Africa: A 12-month review in University of Maiduguri Teaching Hospital, Nigeria	Nigeria	Abstract	PASCAR conference, Dakar 15–20 May, 2013	17
9	Multiple causes (DCM, CAD, RHD)	Thiam I, *et al*.	La mort subite cardio-vasculaire au Sénégal- Etude rétrospective sur 7 ans	Senegal	Abstract	PASCAR conference, Dakar 15–20 May, 2013	235
10	LQTS	Leye M, *et al*.	QT long congenital syncopal évocateur de Syndrome de Jervell Lange Nielsen	Senegal	Abstract	PASCAR conference, Dakar 15–20 May, 2013	1
11	NCCM	Kamotho C, *et al*.	A rare presentation of non-compaction cardiomyopathy in Kenya	Kenya	Abstract	PASCAR conference, Dakar 15–20 May, 2013	1
12	ARVD/C	Kouakam C	Syncope in a black African with arrhythmogenic right ventricular dysplasia	Cameroon	Unpublished data	Unpublished data	1
13	Hypertensive CMP (mainly)	Akinwusi PO, *et al.*	Pattern of sudden death at Ladoke Akintola University of Technology Teaching Hospital, Osogbo, south-west Nigeria	Nigeria	Article	*Vascular Health Risk Management* 2013	Approx 16

BrS: Brugada syndrome, HCM: hypertrophic cardiomyopathy, CAD: coronary artery disease, PASCAR: Pan-African Society of Cardiology, LQTS: long QT syndrome, RHD: rheumatic heart disease, DCM: dilated cardiomyopathy, ARVD/C: arrhythmogenic right ventricular dysplasia/cardiomyopathy, CMP: cardiomyopathy.

**Fig. 1.**
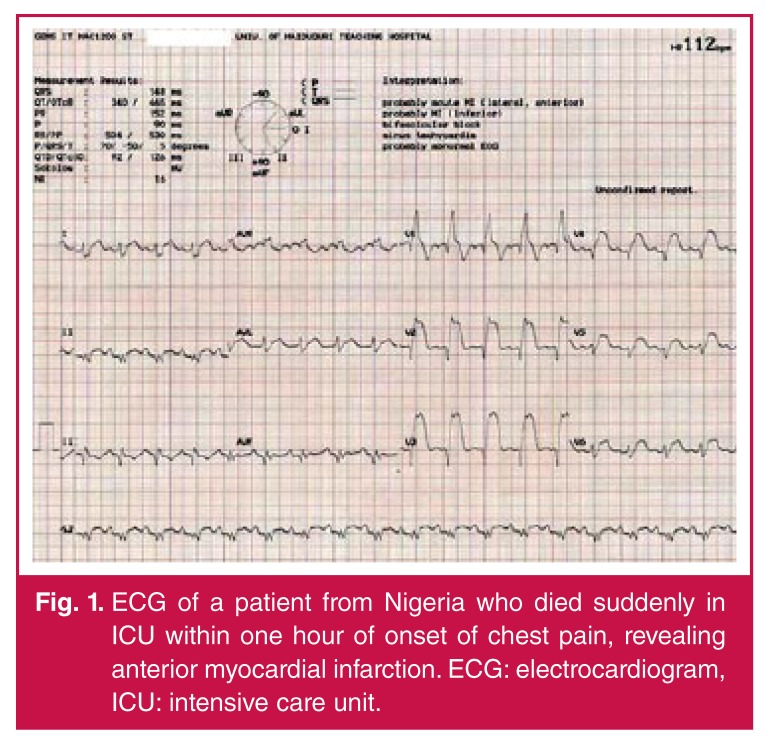
ECG of a patient from Nigeria who died suddenly in ICU within one hour of onset of chest pain, revealing anterior myocardial infarction. ECG: electrocardiogram, ICU: intensive care unit.

SCA from acute myocardial infarction is a rapidly growing cause of morbidity and mortality among black Africans (Fig. 1). However, studies specifically targeted to provide robust data regarding the epidemiology of SCD in Africa are warranted. Missed diagnosis rather than misdiagnosis is a characteristic of unexpected cardiac death in Africa.^31^

**Fig. 2.**
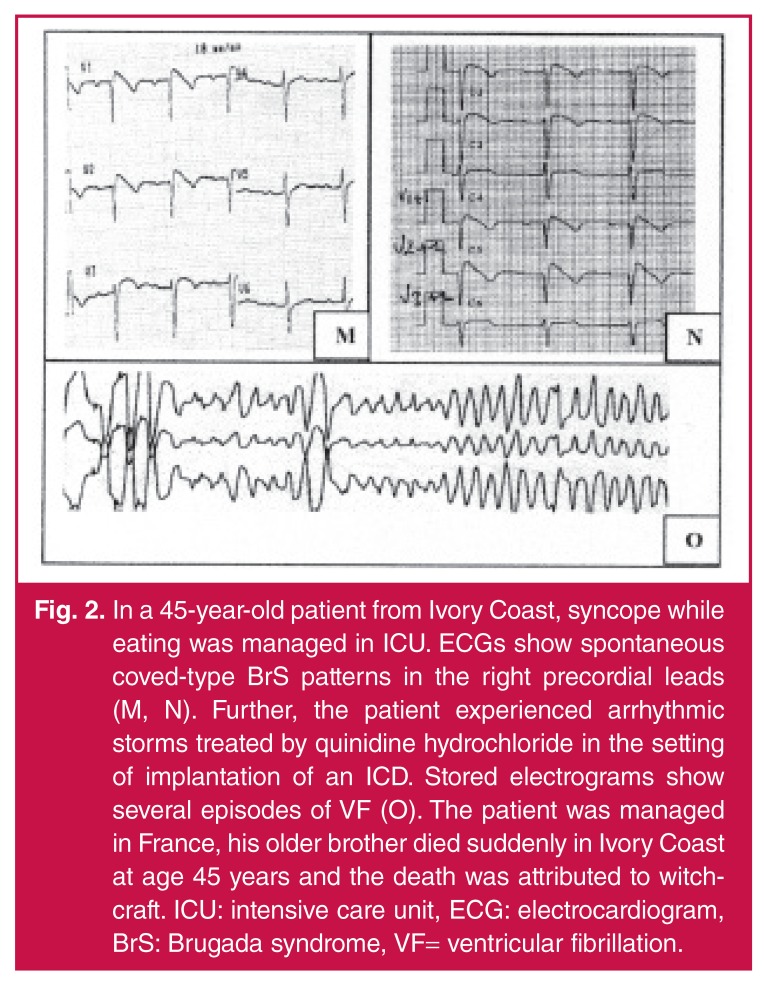
In a 45-year-old patient from Ivory Coast, syncope while eating was managed in ICU. ECGs show spontaneous coved-type BrS patterns in the right precordial leads (M, N). Further, the patient experienced arrhythmic storms treated by quinidine hydrochloride in the setting of implantation of an ICD. Stored electrograms show several episodes of VF (O). The patient was managed in France, his older brother died suddenly in Ivory Coast at age 45 years and the death was attributed to witchcraft. ICU: intensive care unit, ECG: electrocardiogram, BrS: Brugada syndrome, VF= ventricular fibrillation.

The occurrence of an unexpected death in a young, otherwise healthy individual is a devastating event for the family and society. It is now clear that a genetic predisposition may exist and therefore a targeted diagnostic work-up is required in subjects resuscitated from cardiac arrest who show a structurally intact heart. Since these approaches are often not available within the medical system in sub-Saharan Africa and as the population is not aware of the role of the heart in death, juvenile cases are still often attributed in several instances to witchcraft, which prevents the investigation of the medical causes (Fig. 2).^36^, ^41^

The second major concern regarding SCD in Africa is the lack of qualified personnel to accurately diagnose and manage CVD, as well as the absence of basic diagnostic tools in many health facilities.^42^ Given these weaknesses, policies for prevention and control of CVD are incomplete without addressing the problem of SCD. Moreover, there is an opportunity to address these challenges through primary prevention of SCD, secondary prevention through the introduction of widespread cardiopulmonary resuscitation (CPR) education efforts, and lastly, tertiary prevention through treatment of reversible causes, as well as the dissemination of implantable automatic cardiac defibrillators. Understanding the epidemiology of SCD allows the introduction of a comprehensive strategy and implementation of appropriate actions in the WHO global agenda for the fight against NCDs.

The Pan-African Sudden Cardiac Death (Pan-African SCD) study is a collaborative study that aims to collect comprehensive data on the prevalence and incidence of sudden cardiac death in Africa, disease and patient characteristics, mechanisms of cardiac arrest, as well as the survival rate in the setting of underutilised CPR programmes.

## Methods

This is a multicentre, community-based, prospective cohort registry monitoring all cases of SCA. Several countries from all African regions will take part in this Pan-African registry. The specific objectives of the registry are as follows:

• to estimate incidence of SCD in Africa
• to estimate the prevalence of SCD among all causes of death
• to determine factors associated with SCA in African victims
• to study the characteristics and outcomes of SCA in Africa
• to evaluate the extent of the use of cardio-pulmonary resuscitation efforts.

To be eligible, subject must be a resident of the administrative area (district) included in the study registry, and the district must have an updated population census. Inclusion criteria are victims of cardiac arrest, either SCD or aborted SCA. Exclusion criteria are age ≤ 15 years, and refusal of consent (by the family).

## Data collection

In each country and each city of interest, we will conduct a pilot survey in some districts aiming to determine the number of SCDs as well as to evaluate the adherence of the team of each district to effectively collect data during the run-in period of three months. The choice of these districts will be based on eliminating areas of < 30 000 inhabitants in the first step, and random sampling among the remaining districts in the second. We will calculate the approximate mean incidence rate of SCD from all studied districts, taking into account the accuracy of data collection in each area.

After the exclusion of districts with inaccurate data collection, we will randomly select a few districts to constitute a sampling of 150 000 to 200 000 inhabitants in each country. All administrative staff and community healthcare committees of selected districts will be a key component of the research team, helping to identify every case of death in the monitored area.

• Every case of death will be reported by the non-medical district staff in a specific questionnaire form (Appendix 1).
• A nurse will collect circumstances of death to rule out the cause of death, either natural or not.
• A post-graduate medical student will collect socio-demographic and clinical data of every natural death victim.
• A senior physician will study every case of suspected sudden death, using all medical files available, as well as information from the surroundings. Final diagnosis will be obtained by at least two physicians. In the case of disagreement between the experts, a third opinion will be sought. Data will be recorded in an electronic case report form (e-CRF, Appendix 2).
• For victims with ascertained diagnosis of SCD, informed consent will be given to families for autopsy and eventually for genetic analysis.
• Biological sampling will be performed as much as is feasible including: (1) blood sample for genetic test (DNA analysis) of surviving individuals < 40 years in the absence of clinical diagnosis of cardiac disease, or in first-degree relatives of individuals who died suddenly at a young age (< 40 years) and in whom autopsy was either not performed or failed to identify the cause of death, and (2) autopsy for macroscopic and cytological evaluation of victims.

During the time frame of the survey, all residents of districts of interest will be monitored with regard to occurrence of death. The non-medical staff will identify and report each case of death to the medical staff. In addition to the deaths noted by the district administrator’s staff and collaborators, we will pay attention to all notices of deaths in local newspapers, radio, interrogation of health area residents and death certificates from local medical centres. We will propose ECG screening and a long-term follow up for all first-degree relatives of victims under 40 years old.

The Pan-African SCD investigators will be members of the PASCAR task force on SCD, which will be governed by the PASCAR governing council to whom annual progress reports will be provided. During bi-annual meetings, each research team will discuss all cases of suspected SCD. Research teams for each district will be composed of medical and non-medical staff. Both will collect data of the victims.

The role of each member of the research team will as follows:

• The district administrator and other collaborators (civil society volunteers living in the neighbourhood constituting areas of interest) will provide information about all deaths among residents in the area where the survey is conducted.
• One qualified nurse will organise collection of in-hospital deaths. He or she will be expected to work in collaboration with the chief of the mortuary to record out-of-hospital deaths, and with medical students who are voluntarily involved in the project. All cases of SCD will be recorded in the study data book. Either the nurse or the chief of the mortuary will call the student when a suspicion of SCD needs to be investigated within a short period of time, when family members may provide valuable information.
• One post-graduate fellow focusing on the topic ‘SCD epidemiology in Africa’ for his/her thesis will participate in the project. Apart from impromptu visits that will be dictated by certain cases, the student will be expected to visit the mortuary and the chief of the district bimonthly to evaluate the records of all victims and determine causes of death. He or she will be required to review the progress of the work with the supervising physician on a monthly basis, during which the work will be appraised.
• The physician will be the leading coordinator in all districts of the city where the survey is going on. Together with other members of the research team, he will analyse all deaths and resolve uncertainties in suspected cases of SCD.

Approval from the national ethics committee and local institutions will be obtained before starting the survey in each country. Before sampling, informed consent will be obtained from the victim (for survivors of SCA) or his/her legal representative and family members (for SCD) prior to inclusion in this study. Confidentiality will be ensured in accordance with the Helsinki Declaration.

## Statistical analysis

Given the rate of incidence of SCD in the Maastricht study,43 the need for about 70 SCAs to reach statistical significance, and the average sample of 150 000 residents per district randomly chosen, the number of SCAs found during the run-in period will determine the duration of monitoring in every district (and country).

Characteristics of persons experiencing SCA will be presented as frequencies or mean values with standard deviations. Differences between men and women will be tested by chi-square tests for categorical variables and t-tests for continuous variables. Age- and gender-specific incidence rates of ascertained SCA will be calculated. The counts of SCA will be used as the numerators, and the denominators will be the population of all districts included in the survey, as determined by the last census. The rates will be adjusted directly to the age distribution of the total population of each country.

Standard errors and 95% confidence intervals (CI) around the point estimates will be calculated, assuming a Poisson distribution. A Poisson regression model will be used to examine the temporal trends in the incidence of SCA, with categorical year variables and adjustment for age.

Results will be summarised by presenting the relative risk (RR) of SCA for men and women in each year group. Logistic regression models will be used to examine the association between occurrence of SCA and socio-demographic and clinical factors. In the model, year will be modelled categorically, and a non-linear effect of age will be assessed by testing the quadratic term. Comparison of time trends across age groups will be accomplished by including interaction terms between year groups and age.

A value of *p* = 0.05 will be selected for the threshold of statistical significance, except when an interaction will be tested for, when *p* = 0.10 will be used. All analyses will be replicated in 1 000 random samples to ensure that results are robust.

## Discussion

The Pan-African SCD study is the first attempt to comprehensively characterise the SCD burden in Africa. We will collect detailed clinical data as well as information from an electrocardiogram (if available). Other diagnostic evaluations of SCD will also be used where possible. We will, therefore, have a contemporary dataset on the incidence, aetiology, patient characteristics and outcomes of cardiac arrest in African countries.

Almost all causes of SCD have already been reported in blacks living in Africa. However, these data are limited to case or series reports. The PASCAR study will therefore provide a comprehensive data on the epidemiology of SCD in this part of the world. In line with its international outlook, the Pan-African SCD study aims to provide an avenue for examining regional similarities and differences in clinical features, management and outcomes of SCA.

## Strengths and study limitations

The Pan-African SCD study is a community-based, prospective registry aimed at determining the incidence as well as prevalence of SCD in Africa. Although the term ‘sudden cardiac death’ is well defined clinically^7^,^8^ and easily applicable in clinical practice, underlying aetiologies of cardiac arrest have to be ruled out in order to establish a more accurate epidemiological profile.

However, in the developing world, the lack of complex and expensive cardiovascular diagnostic tools, such as exercise ECG testing, stress echocardiography, radionuclide imaging, coronary CT scan and coronary angiography underestimate the ischaemic heart disease burden, which is the leading cause of SCD in developed Western countries.^7^,^8^,^14^ Also, the underutilisation of genetic screening, diagnostic drug challenges and electrophysiological studies in the regions where the study will be conducted are likely to limit the identification of some complex diagnoses, such as those of inherited arrhythmogenic disorders, which are among the commonest causes of SCD in young people under 35 years old.^44^-^46^ Hence, our results will address a global view of SCD burden rather than show the real burden of each aetiology.

## Perspectives

Expected results of this survey are aimed at understanding whether or not SCA is a public health problem in Africa. As preliminary reports tend to indicate, and in the light of what is being done in Western and Asian countries,^47^,^48^ this maiden survey in the field of SCD in Africa will present the platform for advocating preventive public health policies in the fight against SCA, and also primary cardiovascular prevention in general.
